# Working Memory, Fluid Reasoning, and Complex Problem Solving: Different Results Explained by the Brunswik Symmetry

**DOI:** 10.3390/jintelligence9010005

**Published:** 2021-01-21

**Authors:** André Kretzschmar, Stephan Nebe

**Affiliations:** 1 Department of Psychology, University of Zurich, Binzmuehlestrasse 14/7, CH-8050 Zurich, Switzerland; 2 Zurich Center for Neuroeconomics, Department of Economics, University of Zurich, Bluemlisalpstrasse 10, CH-8006 Zurich, Switzerland; stephan.nebe@econ.uzh.ch

**Keywords:** Brunswik symmetry, bandwidth-fidelity dilemma, working memory, reasoning, complex problem solving, intelligence, measurement, structural equation modeling

## Abstract

In order to investigate the nature of complex problem solving (CPS) within the nomological network of cognitive abilities, few studies have simultantiously considered working memory and intelligence, and results are inconsistent. The Brunswik symmetry principle was recently discussed as a possible explanation for the inconsistent findings because the operationalizations differed greatly between the studies. Following this assumption, 16 different combinations of operationalizations of working memory and fluid reasoning were examined in the present study (*N* = 152). Based on structural equation modeling with single-indicator latent variables (i.e., corrected for measurement error), it was found that working memory incrementally explained CPS variance above and beyond fluid reasoning in only 2 of 16 conditions. However, according to the Brunswik symmetry principle, both conditions can be interpreted as an asymmetrical (unfair) comparison, in which working memory was artificially favored over fluid reasoning. We conclude that there is little evidence that working memory plays a unique role in solving complex problems independent of fluid reasoning. Furthermore, the impact of the Brunswik symmetry principle was clearly demonstrated as the explained variance in CPS varied between 4 and 31%, depending on which operationalizations of working memory and fluid reasoning were considered. We argue that future studies investigating the interplay of cognitive abilities will benefit if the Brunswik principle is taken into account.

## 1. Introduction

The question of how complex problem solving (CPS) skills are to be integrated into the nomological network of intellectual abilities was and still is one of the most examined questions in CPS research (e.g., Dörner and Funke 2017; Dörner et al. 1983; Funke and Frensch 2007; Kretzschmar et al. 2016; Süß 1996; Süß and Kretzschmar 2018). While most studies have investigated the association between (subconstructs of) intelligence and CPS (for an overview, see Stadler et al. 2015), there are only a few studies that have additionally considered working memory as a relevant cognitive ability to solve complex problems (for an overview, see Zech et al. 2017).

Whereas there is a consensus regarding the high correlation between intelligence and CPS (see, e.g., Kretzschmar et al. 2016; Stadler et al. 2015), the results regarding the effect of working memory on CPS are inconsistent. For example, in the studies of Wittmann and Süß (1999) and Greiff et al. (2016), both working memory and (subconstructs of) intelligence significantly explained variance in CPS. However, in the study of Bühner et al. (2008), only working memory but not fluid reasoning significantly explained CPS variance if both abilities were considered. The opposite was found in the study of Süß and Kretzschmar (2018), in which only fluid reasoning but not working memory significantly explained variance in CPS. Zech et al. (2017) argued that such inconsistent findings could be explained by different aggregation (or generalization) levels, as well as different task contents (i.e., verbal, numerical, figural), of the operationalizations used. In detail, Zech et al. (2017) empirically demonstrated—based on a CPS measure with high demands on figural-numerical contents—that only fluid reasoning but not working memory significantly explained CPS variance if aggregated (i.e., content-unspecific, based on several different content operationalizations) or numerical operationalizations were applied; only working memory but not fluid reasoning significantly explained CPS variance if figural operationalizations were used; and both working memory and fluid reasoning significantly explained (different aspects of) CPS variance if verbal operationalizations were considered. Thus, the study clearly provided evidence that different operationalizations of the same constructs can lead to different empirical associations. These findings are also in line with research showing that considering content factors (i.e., verbal, numerical, figural) in addition to cognitive operations (e.g., fluid reasoning, memory) based on a faceted model (Süß and Beauducel 2005) represents the structure of cognitive abilities very well (e.g., Oberauer et al. 2003; Süß and Beauducel 2015). Therefore, as Zech et al. (2017) concluded, the key issue with regard to the interpretation of empirical findings is to consider an adequate match between different operationalizations, or, in other words, to take the Brunswik symmetry principle Wittmann (1988) into account.

### 1.1. The Brunswik Symmetry Principle

**Figure 1:**
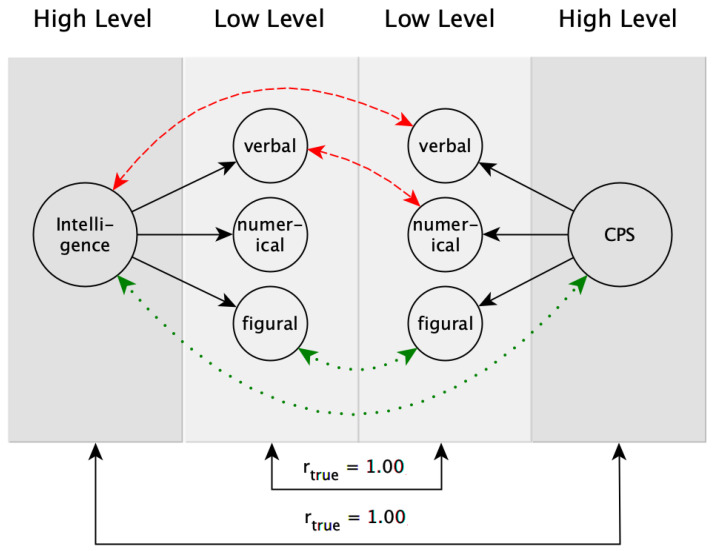
Illustration of the Brunswik symmetry principle according to Wittmann (1988). Dotted (green) lines = symmetrical (fair) comparison; dashed (red) lines = asymmetrical (unfair) comparison.

Wittmann (1988) developed the Brunswik symmetry principle as an adaption of Brunswik’s lens model Brunswik (1955). The Brunswik symmetry principle can be used to describe and explain the association between hierarchically organized constructs at different levels of aggregation (or generalization). A hierarchically organized construct is understood as a multidimensional construct, which includes subconstructs of different levels of specificity on the respective hierarchical levels. For example, based on contemporary models of intelligence, such as the Cattell–Horn–Carroll (CHC) theory McGrew (2009), the construct of intelligence contains three hierarchical levels (or levels of generalization). General intelligence (*g*) is considered to be the highest level (i.e., Stratum III in CHC theory), whereas fluid reasoning or short-term memory as more specific abilities are considered to be at the next lower level (i.e., Stratum II), and quantitative reasoning or deductive reasoning are considered to be the most specific abilities at the lowest level (i.e., Stratum I). The basic idea of the Brunswik symmetry principle is that a true correlation between two hierarchically organized constructs is unbiasedly represented by the empirically observed correlation if and only if (a) the applied measurements correspond to the intended level of generalization (e.g., fluid reasoning as a broad ability should be operationalized with verbal, figural, and numerical task contents, and not only with figural task contents, which would be appropriate as an operationalization for figural fluid reasoning as a more specific, narrow ability; see, e.g., Gignac 2015; Wilhelm 2005), and (b) the chosen levels of generalization are similar, which means symmetrical, for both constructs (i.e., a broad operationalization of a cognitive ability corresponds best to a broad operationalization of another cognitive ability, and a narrow operationalization corresponds best to another narrow operationalization with similar task contents). Consequently, an observed correlation underestimates the true correlation between two hierarchically organized constructs if operationalizations from different levels of generalization or with dissimilar task contents are correlated. Figure 1 illustrates the idea of the Brunswik symmetry principle.

For the sake of simplicity, let us assume that the two constructs intelligence and CPS are perfectly correlated (i.e., *r_true_* = 1.00). Accordingly, the observed correlation will be highest if the operationalizations are at the same aggregation level and have similar requirements regarding the contents (green/dotted lines in Figure 1). In this case, a symmetrical (fair) comparison of the two constructs is conducted, and—regardless of whether this comparison is conducted on a high or low level of aggregation—the observed correlation is an unbiased representation of the true correlation between intelligence and CPS. However, if operationalizations at different aggregation levels (upper red/dashed line in Figure 1) or operationalizations at the same aggregation level but with different content requirements (lower red/dashed line in Figure 1) are used, an asymmetrical (unfair) comparison is conducted. In this case, the observed correlation will be attenuated. As summarized by Kretzschmar et al. (2018), this effect is mainly caused by different construct representations (i.e., whether the operationalization covers the constructs in its entire breadth or only partial aspects of it) and different reliabilities of aggregation levels (i.e., aggregation usually leads to higher reliability, which sets the boundary to validity estimations; see Wittmann 1988).

**Figure 2:**
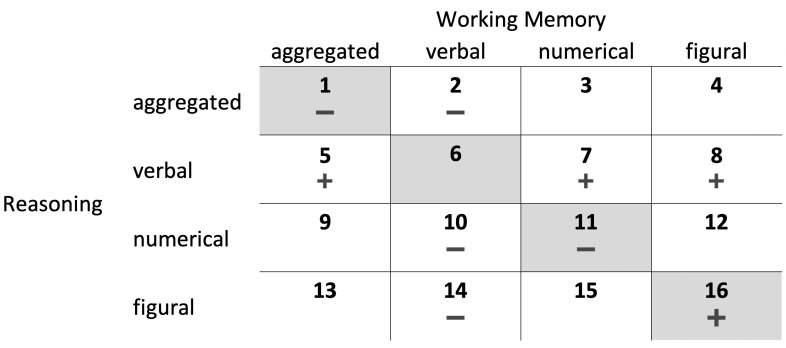
Conditions numbered from 1 to 16 as combinations of different aggregation levels and contents of the operationalizations investigated in the present study (each condition with two models; see Figure 3). Gray-shaded conditions were examined in Zech et al.’s (2017) study and represent symmetrical (fair) combinations in terms of the predictor-predictor symmetry as similar operationalizations of working memory and fluid reasoning were considered. The other conditions can be considered as potentially asymmetrical (unfair) in terms of the predictor-predictor symmetry as different aggregation levels or contents were considered. Ad-hoc expectations whether working memory incrementally explains variance above and beyond fluid reasoning (+) or not (−) are displayed for each condition.

Several studies have empirically demonstrated the usefulness of the Brunswik symmetry principle in diverse research areas^1^, for example, studies investigating the correlation between non-cognitive personality traits and intelligence (e.g., Kretzschmar et al. 2018; Rammstedt 2018), the association between different cognitive abilities (e.g., Kretzschmar et al. 2017; Redick et al. 2016; Wittmann and Hattrup 2004), the prediction of academic performance with cognitive and non-cognitive personality traits (e.g., Coyle et al. 2015; Kretzschmar et al. 2016; Spengler et al. 2013), and the prediction of occupational and other behavioral criteria with personality traits (e.g., Figueredo et al. 2016; Paunonen and Ashton 2001; Ziegler et al. 2014). To the best of our knowledge, Zech et al. (2017) is the only study in which the relations between working memory, fluid reasoning, and CPS were investigated by systematically considering different aggregation levels and contents of the operationalizations in terms of the Brunswik symmetry principle. However, Zech et al. (2017) only considered combinations of working memory and fluid reasoning operationalizations with the same content (e.g., figural working memory and figural fluid reasoning; gray-shaded conditions in Figure 2) but not with different contents (e.g., verbal working memory and figural fluid reasoning; non-shaded conditions in Figure 2). In addition, measurement error was not controlled for in Zech et al.’s (2017) study, which makes it difficult to compare the results across conditions.

In summary, although Zech et al. (2017) provided an important impulse for research into the relations between working memory, fluid reasoning, and CPS considering the Brunswik symmetry principle, a more comprehensive view is necessary to understand the relations of these cognitive constructs and the impact of the Brunswik symmetry principle.

### 1.2. The Present Study

The present study had two aims. First, we wanted to conceptually replicate Zech et al.’s (2017) finding findings regarding the association between working memory, fluid reasoning, and CPS based on different measurements. In detail, we were interested in whether working memory incrementally explains variance in CPS above and beyond fluid reasoning. According to the findings of Süß and Kretzschmar (2018), the CPS measure used in the present study put similar demands on the content as the CPS measure in Zech et al.’s (2017) study, that is, strong requirements concerning figural content, to a slightly lesser extent requirements concerning numerical content, and only weak requirements concerning verbal content. Therefore, it can be assumed that the findings of the present study would be consistent with those of the conditions considered in Zech et al.’s (2017) study.

Second, we wanted to investigate whether it is possible to predict results with the help of the Brunswik symmetry principle. To do so, we systematically manipulated the symmetrical match with regard to aggregation levels and contents of the operationalizations. In detail, we considered 16 combinations of operationalizations (i.e., four different operationalizations each for working memory and fluid reasoning: verbal, numerical, and figural content, as well as an aggregated measure, over all three content types), which were used to explain variance in CPS (see Figure 2). Following the Brunswik symmetry principle, the highest association between the operationalizations should be observed in a symmetrical (fair) condition between working memory, fluid reasoning, and CPS.

As three constructs were investigated in this study, the Brunswik symmetry principle can have an effect in two different ways. The first type, which we call predictor-criterion symmetry from here on, is about a symmetrical (fair) match between predictors (i.e., working memory and fluid reasoning) and the criterion (i.e., CPS). For example, as the CPS operationalization used in the present study put strong requirements on figural and numerical content and weak demands on verbal content, a condition in which figural and/or numerical operationalizations of both working memory and fluid reasoning were applied can be considered a symmetrical (fair) comparison. Conditions in which the operationalizations of working memory and fluid reasoning did not match the content requirements or aggregation level of the CPS operationalization can be considered asymmetrical (unfair), whereby a condition with only verbal operationalizations of the predictors can be considered the most asymmetrical. The predictor-criterion symmetry can be evaluated on the basis of the explained variance of the criterion: The higher/lower the explained CPS variance, the more symmetrical/asymmetrical the comparison is.

The second type, hereinafter referred to as predictor-predictor symmetry, refers to the similarity of operationalizations of the two predictors to each other. If operationalizations with the same content requirements or aggregation levels are used for working memory and fluid reasoning (e.g., numerical operationalizations for each), then this can be considered a symmetrical (fair) condition. However, if operationalizations with different content requirements or aggregation levels are used (e.g., aggregated working memory and verbal fluid reasoning), then this is considered an asymmetrical (unfair) condition in which either working memory or fluid reasoning is favored, depending on which has a better match to the content requirements of the criterion. For example, this would mean here that a verbal operationalization of one predictor and a figural or numerical operationalization of the other predictor would lead to a systematic discrimination of the former (i.e., underestimation of its relation with the criterion). Therefore, in terms of the predictor-predictor symmetry, only comparisons with similar operationalizations of the predictors can be considered as symmetrical (fair).

In summary, the following expectations were derived based on the Brunswik symmetry principle in combination with Zech et al.’s (2017) findings. The first two aspects relate to the question whether and under which conditions working memory explains CPS variance above and beyond fluid reasoning. The third aspect relates to the question of the most symmetrical (fair) match, that is, whether different operationalizations represent differently symmetrical matches.

1. With regard to the first aim of the study (i.e., replication of previous findings) and, thus, according to Zech et al.’s (2017) results, working memory does not incrementally explain variance in CPS above and beyond fluid reasoning if aggregated (i.e., content-unspecific based on all three content operationalizations; condition 1 in Figure 2) or numerical (condition 11) operationalizations were applied. Furthermore, working memory incrementally explains variance in CPS above and beyond fluid reasoning if figural operationalizations were considered (condition 16). We had no expectations regarding verbal operationalizations (condition 6) as Zech et al.’s (2017) study provided different findings with regard to different CPS aspects, which were not considered in the present study (see below).
2. With regard to the second aim of the study and in terms of the predictor-predictor symmetry (i.e., considering combinations of different aggregation levels and contents of the operationalizations of the predictors), we expected an asymmetrical (unfair) comparison if a verbal operationalization was combined with any other operationalization as the CPS measure used in the present study had only weak requirements concerning verbal contents. In detail, aggregated (condition 5), numerical (condition 7), and figural (condition 8) working memory should incrementally explain CPS variance above and beyond verbal fluid reasoning. Consequently, verbal working memory should not incrementally explain CPS variance above and beyond aggregated (condition 2), numerical (condition 10), and figural (condition 14) fluid reasoning. We had no specific expectations regarding the other conditions (i.e., 3, 4, 9, 12, 13, and 15). As figural and numerical abilities are rather highly correlated, their interaction within an aggregated operationalization and their relation to an aggregated operationalization is difficult to predict.^2^
3. With regard to the CPS measure used in the present study and combinations of the same content (i.e., conditions 1, 6, 11, and 16), a symmetrical (fair) comparison in terms of the predictor-criterion symmetry would be based on figural and numerical operationalizations of working memory and fluid reasoning (as the CPS measure had only weak requirements regarding verbal content). Given equal reliability across all conditions, it means the highest proportion of CPS variance should be explained based on figural working memory and fluid reasoning operationalizations (condition 16), followed by numerical operationalizations of both constructs (condition 11). Verbal operationalizations should explain the least variance in CPS (condition 6). Aggregated operationalizations (condition 1) should explain more CPS variance than verbal operationalizations but it is unclear whether less (due to the irrelevant verbal aspect) or equal/more (due to the combination of figural and numerical aspects) CPS variance than either figural or numerical operationalizations alone. As outlined above, we had no specific expectation in terms of the predictor-criterion symmetry regarding the other conditions combining figural and numerical contents.

## 2. Materials and Methods

The present study used the freely available data set of Kretzschmar and Süß (2015). In the following, only those operationalizations are described which are relevant to the research question at hand. For a complete description of all operationalizations, see Kretzschmar and Süß (2015) and Süß and Kretzschmar (2018). Please note that condition 1 of the present study (see Figure 2) was investigated in a modified form in Süß and Kretzschmar (2018) in the context of a broader research question regarding the influence of knowledge and cognitive abilities on CPS performance and based on the same data set. Although the analysis strategy in both studies differs in some crucial points (i.e., with regard to the consideration of the measurement error, different calculations of the CPS score, and including further variables irrelevant for the present research question), the results concerning condition 1 are presented here mainly for the sake of completeness. A systematic investigation of the Brunswik symmetry principle based on the other 15 conditions as the main aim of the present study is a novel and as yet unexamined research question. As the present study is based on an already used data set, we consider the present study as exploratory (Thompson et al. 2020), which is addressed in more detail in the Discussion section.

### 2.1. Participants

The full data set consists of 159 participants, from which seven non-native German speakers were excluded for the analysis due to the high language requirements of the ability tests. Participants of the final sample (*N* = 152) had a mean age of 23.99 (*SD* = 4.43) years. All participants were university students as in previous studies (e.g., Wittmann and Süß 1999; Zech et al. 2017). Gender was equally distributed.

### 2.2. Material

#### 2.2.1. Working Memory

Three tasks from the computerized working memory test battery by Oberauer et al. (2003) were used. The figural dot span task (adaptive version; sometimes named spatial coordination) primarily measured the coordination function, whereas the numerical memory updating task (adaptive version) and the verbal reading span task (non-adaptive) primarily measured the storage and processing function of working memory. All working memory tasks can be considered as speeded power tasks as participants had to answer within a certain time frame. Each of the three task scores was z-standardized. The aggregated working memory score was calculated as the average of these task scores.

#### 2.2.2. Fluid Reasoning

Selected tasks of the Berlin Intelligence Structure (BIS) test (Jäger et al. 1997) measuring fluid reasoning and processing speed were applied. For the present study, we only considered the nine fluid reasoning tasks as processing speed showed only weak or no associations with CPS in the present study (see Süß and Kretzschmar 2018), as well as in previous studies (see, e.g., Kretzschmar et al. 2016; Süß 1996). In line with the test instruction, the fluid reasoning tasks were completed under time constraints and, thus, can be considered as speeded power tasks, as well. All nine task scores were z-standardized. In each case, three task scores were averaged in order to obtain a content-specific score for verbal, numerical, and figural fluid reasoning, respectively. Following the standard procedure of the BIS test, the aggregated fluid reasoning score was calculated based on three content-balanced parcels (for further details, see, e.g., Süß and Beauducel 2015).

#### 2.2.3. Complex Problem Solving

The computer-based measurement FSYS (Wagener 2001) was used. FSYS is based on Dörner’s (1986) theoretical framework regarding the assessment of CPS. According to Süß and Kretzschmar (2018), FSYS can be classified as a complex real-life-oriented system (also named microworld in CPS research) in distinction to complex artificial systems. The goal of FSYS is to manage five independent forests to increase the financial value of the forest enterprise. In order to do so, 85 variables connected via linear, exponential, or logistic relations have to be monitored or manipulated. Following the standard procedure of CPS assessment, participants received an introduction including a non-evaluated exploration phase before the actual control phase was completed (Kretzschmar et al. 2017). Participants were asked to finish the control phase within 90 min; thus, FSYS can also be considered as a speeded power test. We used the SKAPKOR scale (ranging between 0 and 100 with higher scores representing a better CPS performance) which is based on the forest enterprise’s total capital after 50 simulated months as the CPS performance indicator (see Wagener 2001).^3^ Previous studies provided evidence regarding the validity of FSYS, in particular, with regard to educational (Stadler et al. 2016) and occupational (Wagener and Wittmann 2002) achievements.

In addition to the CPS control performance, the acquired knowledge about the CPS system is often considered as a further CPS indicator (e.g., Fischer et al. 2012). Therefore, Wagener’s (2001) FSYS knowledge test was used to assess the knowledge acquired during the 50 simulated months. The 11 multiple-choice items (dichotomous scoring) cover heterogeneous aspects of the system with regard to system and action knowledge. We used the average test score across all items.

Previous research has shown that the correlation between the CPS performance and knowledge indicators is relatively high (see, e.g., Goode and Beckmann 2010; Greiff et al. 2013). Therefore, the empirical distinction between knowledge acquisition and control performance as separate CPS processes was critically questioned from a psychometric (e.g., Kretzschmar et al. 2017), as well as from a criterion validity (Kretzschmar 2015), perspective. Hence, for the present study and in line with previous research (e.g., Kretzschmar et al. 2014; Mainert et al. 2015; Rudolph et al. 2018), we used an averaged total CPS score based on the z-standardized control and knowledge scores.^4^

### 2.3. Procedure

The assessment was split into two sessions, each lasting about 2.5 h. Working memory and fluid reasoning were assessed in the first session, whereas CPS and other, for the present study irrelevant, constructs were assessed in the second session. The study was originally designed as a training study; thus, the time between the two sessions varied between one day and one week (for further details of the study design, see, Kretzschmar and Süß 2015). The tests were administered in groups of up to 20 people in computer laboratories. As a compensation for their effort, participants received course credit or could participate in a book raffle. All participants were informed in advance about the content of the study, the voluntary nature of their participation, and data protection issues. All subjects provided informed consent.

### 2.4. Statistical Analysis

As described in the Material section, a total scale score was calculated for each operationalization and for each level of aggregation. Correlations between these scores, as well as their corresponding 95% confidence intervals (CI), were calculated based on 5000 bootstrapped Pearson correlations. Reliability was estimated via McDonald’s *ω* (see, e.g., Dunn et al. 2013) if multiple indicators were available. Reliability estimations were taken from Wagener (2001) and Oberauer et al. (2003) for the CPS performance scale and for the content scores of working memory, respectively.

To control for measurement error of the different operationalizations, we used single-indicator latent variables (see, e.g., Brown 2015) for all analyses. In single-indicator models, latent variables are each defined by one indicator consisting of an equally-weighted composite score (i.e., the manifest mean scale score). The true-score variance for the latent variables is obtained by fixing the unstandardized error of their indicator to (1 − *reliability*) * *s^2^*, where *s^2^* is the sample variance of the composite score.

**Figure 3:**
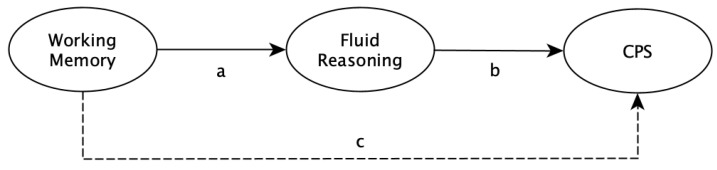
Structural models for all analyses. Model 1 (without dashed line): Complex problem solving (CPS) variance is explained by fluid reasoning, in which variance is explained by working memory. Model 2 (with dashed line): A direct path from working memory to CPS is added to Model 1. Measurement models are omitted for the sake of simplicity.

Based on the single-indicator latent variables, we applied structural equation modeling (SEM) to test whether working memory explains CPS variance above and beyond fluid reasoning. To do so, we first estimated Model 1 as presented in Figure 3. In this model, CPS variance is directly explained by fluid reasoning (path b in Figure 3), in which variance is explained by working memory (path a in Figure 3). In the next step, we estimated Model 2, in which we added a direct path from working memory to CPS in Model 1 (path c in Figure 3). Based on these two models, the incremental explained CPS variance (Δ R^2^) was evaluated based on a hierarchical F test (Cohen et al. 2003, p. 171, formula 5.5.1) with *α* = 0.003 (= 0.05/16; Bonferroni correction). The 95% CIs of the standardized regression weights and of the explained variances were calculated based on 5000 bootstrapped draws. For all models, maximum likelihood (ML) estimation was used. Model fit of Model 1 was evaluated based on standard fit indices and the commonly used cutoff values (e.g., Schermelleh-Engel et al. 2003). Specifically, we used the *χ^2^* goodness-of-fit statistic (*p* > 0.05), Comparative Fit Index (*CFI* ≥ 0.97), Root Mean Square Error of Approximation (*RMSEA* ≤ 0.05), and Standardized Root Mean Square Residual (*SRMR* ≤ 0.05). Model 2 was fully saturated (i.e., with zero degrees of freedom); thus, model fit could not be evaluated.

The sample size was comparable to or larger than those of most previous studies (e.g., Bühner et al. 2008; Wittmann and Süß 1999; Zech et al. 2017) and sufficient for SEM based on single-indicator latent variables in the present study. Following the 10:1 to 20:1 rule of thumb regarding the ratio of estimated parameters to sample size (e.g., Kyriazos 2018), the optimal sample size was between 90 and 180 participants as nine parameters had to be estimated in the most complex model. However, as the sample size was not optimal to investigate weak to moderate correlations (see, Kretzschmar and Gignac 2019), point estimates of correlations should be interpreted only with considering the bootstrapped CIs (Cumming 2013). Furthermore, 23.68% data for the CPS scores were missing. The assumption of missing completely at random (MCAR) seems to be reasonable and was empirically supported based on Little’s (1988) test considering all scale scores and demographic data in the data set: *χ^2^*(22) = 19.88, *p* = 0.59. As methodological studies have shown that missing data methods provide virtually unbiased results for this or even larger amount of missing data under the assumption of MCAR (e.g., Dong and Peng 2013), we used the Full Information Maximum Likelihood (FIML) procedure to account for missing data.^5^ The data are publicly available via the Open Science Framework: https://osf.io/n2jvy. The study was not preregistered and, thus, should be considered as exploratory.

## 3. Results

Table 1 displays the descriptive statistics, reliability estimates, and correlations. Standardized regression weights as labeled in Figure 3 and explained CPS variance for each model are shown in Table 2. All models demonstrated good to very good model fit according to our evaluation criteria, except those of conditions 5, 7, 8, and 14, which are discussed below.

### 3.1. Does Working Memory Incrementally Explain CPS Variance?

With regard to replicating Zech et al.’s (2017) findings, our results were only partly consistent. As in Zech et al.’s (2017) study, working memory did not incrementally explain variance in CPS above and beyond fluid reasoning if aggregated (condition 1 in Figure 2) or numerical (condition 11) operationalizations were applied. However, the same pattern was found for verbal (condition 6) and figural (condition 16) operationalizations, which was not in line with Zech et al.’s (2017) results. These findings were supported by hierarchical F tests, of which none indicated an incremental explanation of CPS variance in Model 2 of these conditions (all *p*s > 0.003).

With regard to the predictor-predictor symmetry and, thus, considering the conditions in which combinations of different aggregation levels and contents were examined, all of our six expectations except for one were confirmed. In detail and regarding verbal fluid reasoning, aggregated ($\Delta R_{adj}^{2}$ = 0.12, hierarchical F test: *p* < 0.001; condition 5) and figural ($\Delta R_{adj}^{2}$ = 0.09, hierarchical F test: *p* <0.001; condition 8) working memory incrementally explained CPS variance above and beyond verbal fluid reasoning as expected. However and against our expectation, numerical working memory did not incrementally explain CPS variance above and beyond verbal fluid reasoning ($\Delta R_{adj}^{2}$ = 0.04, hierarchical F test: *p* = 0.004; condition 7). With regard to verbal working memory and in line with our expectations, verbal working memory did not incrementally explain CPS variance above and beyond aggregated ($\Delta R_{adj}^{2}$ = 0.00; condition 2), numerical ($\Delta R_{adj}^{2}$ = 0.00; condition 10), and figural ($\Delta R_{adj}^{2}$ = 0.03, hierarchical F test: *p* = 0.009; condition 14) fluid reasoning. These findings also explain why the fits of Model 1 in conditions 5, 7, 8, and 14 were not acceptable. Although the hierarchical F-test showed statistically significant incremental variance explanation only in conditions 5 and 8, there was at least a weak correlation between working memory and CPS in all four conditions, which was not adequately considered in these models (see Table 2). With regard to the conditions for which we had no expectations (i.e., conditions 3, 4, 9, 12, 13, and 15), working memory did not incrementally explain CPS variance above and beyond fluid reasoning in any of them (hierarchical F tests: all *p*s > 0.003).

In summary, working memory explained CPS variance above and beyond fluid reasoning in only 2 out of 16 conditions (max. $\Delta R_{adj}^{2}$ = 0.12).

### 3.2. Do Different Combinations Represent Differently Symmetrical Matches?

The results regarding the most symmetrical match in terms of the predictor-criterion symmetry (i.e., indicated by the highest CPS variance explained) demonstrated substantial differences between the conditions. The combination of verbal working memory and figural fluid reasoning (condition 14) showed the numerically highest explanation of CPS variance ($R_{adj}^{2}$ = 0.31). Combinations with verbal fluid reasoning (i.e., conditions 5 to 8) showed the numerically lowest explanation of CPS variance (0.04 $\leq R_{adj}^{2}\leq$0.17). All other combinations showed relatively similar proportions of explained CPS variance (0.23 $\leq R_{adj}^{2}\leq$ 0.28). In addition, our four expectations regarding the most symmetrical match based on combinations of the same content were mostly correct. As expected, verbal operationalizations (condition 6) explained least CPS variance ($R_{adj}^{2}$ = 0.04). Furthermore and in line with our expectations, figural operationalizations (condition 16) showed some of the highest CPS variance explanations ($R_{adj}^{2}$ = 0.27). The CPS variance explained by numerical operationalizations (condition 11) was numerically lower ($R_{adj}^{2}$ = 0.23) but similar to figural operationalizations. Aggregated operationalizations (condition 1) explained more CPS variance ($R_{adj}^{2}$ = 0.26) than the verbal operationalizations and virtually the same proportion as the figural and numerical operationalizations.

In summary, the proportion of explained CPS variance varied between 4 and 31%, depending on which operationalizations of working memory and fluid reasoning were considered. Moreover, the Brunswik symmetry principle seems a valid indicator of which combinations of operationalizations would show the most or least explained variance.

## 4. Discussion

The present study aimed to shed further light on the relations between working memory, fluid reasoning, and CPS, on the one hand, and to empirically evaluate the Brunswik symmetry principle (Wittmann 1988), on the other. By considering 16 different combinations of operationalizations of working memory and fluid reasoning, we found that working memory incrementally explained CPS variance above and beyond fluid reasoning in only two of these conditions. Furthermore, the findings provide clear evidence that different operationalizations of the same constructs can lead to very different results, which can be explained by the Brunswik symmetry principle.

### 4.1. Working Memory, Fluid Reasoning, and CPS

The relation of working memory and intelligence (e.g., Ackerman et al. 2005; Oberauer et al. 2005) and of intelligence and CPS (e.g., Kretzschmar et al. 2016; Stadler et al. 2015) within the nomological network of cognitive abilities have stimulated a great amount of research. However, only a few studies have simultaneously considered all three constructs with inconsistent results. Zech et al. (2017) argued that these inconsistent results can be explained by means of the Brunswik symmetry principle; that is, operationalizations that differ regarding the level of aggregation (or generalization) and task contents (i.e., verbal, numerical, figural) lead to different results with regard to the interplay of working memory, fluid reasoning, and CPS. Extending this idea, we systematically considered four different operationalizations (i.e., aggregated, verbal, numerical, and figural) of both working memory and fluid reasoning. Our results showed that working memory incrementally explained CPS variance only in 2 out of 16 conditions. For both conditions, we expected that working memory should incrementally explain CPS variance due to an asymmetrical (unfair) comparison in terms of the predictor-predictor symmetry. Thus, in these conditions the operationalization of fluid reasoning did not match well the cognitive requirements regarding the content of the CPS operationalization which results in a relatively stronger impact of working memory.

Another interesting finding is that working memory did not incrementally explain CPS variance in any of the conditions that were also considered in Zech et al.’s (2017) study. The most obvious difference between the present study and that of Zech et al. (2017) is in the operationalizations of working memory. While broader operationalizations were used in Zech et al.’s (2017) study, each with several tasks balancing different processes of working memory (see Oberauer et al. 2003), the content-specific operationalizations in the present study consisted of only one task each. Therefore, working memory in Zech et al.’s (2017) study was more representative of the construct (see Shadish et al. 2002) and less task-specific than in the present study. As broader and, thus, more representative operationalizations provide more accurate insights into the relation between different constructs (for an empirical demonstration, see, e.g., Kretzschmar et al. 2016), the findings of the present study are not suitable to generally rule out that working memory incrementally explains CPS variance above and beyond fluid reasoning in some conditions. However, it should be noted that the explanation of the differently broad operationalizations of working memory is not sufficient when one looks at the studies that used comparatively narrow operationalizations of working memory as in the present study and applying a symmetrical (fair) comparison in terms of the predictor-predictor symmetry regarding working memory and fluid reasoning (e.g., Bühner et al. 2008).

Another explanation for the inconsistent results between Zech et al.’s (2017) and the present study refers to the different cognitive requirements of CPS operationalizations. Apart from Wittmann and Süß’s (1999) study, the present study, as well as all other studies on this topic, used one specific CPS operationalization. It may well be that the cognitive requirements associated with working memory differ substantially between the different CPS operationalizations. In order to draw generalizable conclusions on a level of psychological constructs, it is therefore essential for future research to simultaneously consider different CPS operationalizations (Funke et al. 2017).

In summary, the findings of the present study considered on its own indicate that there is little evidence that working memory incrementally explains CPS variance above and beyond fluid reasoning, particularly if a symmetrical (fair) comparison of all operationalizations is considered. In the context of the other studies that examined the relation of working memory, fluid reasoning, and CPS, the present findings fit well into the rather inconsistent picture of empirical results. Whether or not working memory plays a unique part in solving complex problems beyond (subconstructs of) intelligence, therefore, requires further research that necessarily considers broad operationalizations of all three constructs (see, e.g., Wittmann and Süß 1999).

### 4.2. The Brunswik Symmetry Principle and the Choice of Operationalizations

The present study is one of a series of studies (e.g., Kretzschmar et al. 2017; Wittmann and Hattrup 2004; Zech et al. 2017) that have emphasized and empirically demonstrated the importance of the Brunswik symmetry principle (Wittmann 1988). As can be seen from the findings of these studies, the choice of operationalizations in terms of the level of aggregation (or generalization) and breadth of content can have a substantial impact on the empirical findings. This is also relevant, for example, to the debate on the extent to which working memory and fluid reasoning represent different or identical cognitive constructs (e.g., Ackerman et al. 2005; Oberauer et al. 2005). In the present study, the correlations between the two constructs ranged from 0.04 to 0.53 (see Table 1; disattenuated correlation: *r_max_* = 0.73). Depending on the level of aggregation, the present study provides further evidence that both constructs are highly correlated (Oberauer et al. 2005). Therefore, we conclude that the Brunswik symmetry principle adds another layer to the decision-making process when it comes to selecting an adequate operationalization of psychological constructs not only for CPS research (see Flake and Fried 2020).

However, the present study also demonstrated that the Brunswik symmetry principle can guide this decision-making process. An asymmetrical (unfair) comparison, and thus attenuated empirical correlations, can be avoided by considering the best possible match between different operationalizations—either based on considerations of cognitive requirements or on previous research findings (e.g., for a systematic investigation of personality-ability relations, see Kretzschmar et al. 2018). Even if no such information should be available, the Brunswik symmetry principle can be helpful for the choice of operationalization. Broader operationalizations always include narrower operationalizations, so that on the basis of the broad operationalization it can be exploratively investigated (de Groot 2014) which aggregation level represents the more appropriate level of symmetry (for an empirical demonstration, see, e.g., Kretzschmar et al. 2017). The higher time requirement for conducting a study based on broader operationalizations should normally be compensated for by substantially reducing the risk of finding zero or weak empirical associations of actually correlated psychological constructs because of choosing a too narrow operationalization out of common practice or unawareness. For example, Raven’s Matrices tests (Raven et al. 1998) or similar measurements of figural fluid reasoning are often used as the only operationalization and also as one of the best indicators of general intelligence (*g*). This practice is not only based on questionable assumptions regarding the operationalization of *g* (see, e.g., Gignac 2015; Lohman and Lakin 2011; Süß and Beauducel 2015) but is also very likely to lead to biased results (for an empirical demonstration regarding the construct validity of cognitive abilities, see, e.g., Kretzschmar et al. 2016). Therefore, in case of uncertainty about which operationalization to choose, one is on the safe side in terms of the Brunswik symmetry principle if (too) broad operationalizations are applied (and then different aggregation levels are investigated).

In summary, on the one hand, Zech et al.’s (2017) conclusion that the Brunswik symmetry principle should be taken into account when interpreting the results within, as well as between, different studies can be explicitly endorsed, whereby greater significance should be attributed to those studies that are based on symmetrical (fair) comparisons. On the other hand, as outlined above, considering the Brunswik symmetry principle is also helpful and necessary in designing studies when selecting the appropriate operationalizations.

### 4.3. Limitations and Future Research

The findings of the present study need to be interpreted in light of some limitations. First, participants were recruited from the subpopulation of university students, which generally has above-average cognitive abilities. As such range restrictions usually result in reduced correlations, the associations between the cognitive constructs were most likely underestimated. Therefore, the presented results should be interpreted with caution in terms of the general association between working memory, fluid reasoning, and CPS.

Second, as Wilhelm and Schulze (2002) emphasized, investigating cognitive constructs with differently strong time restrictions can lead to biased correlations due to varying degrees of variance in mental speed. For example, if working memory tasks have strong time constraints but the fluid reasoning and CPS measurements have less time constraints, then the different time constraints alone result in a lower correlation between working memory and CPS compared to the correlation between fluid reasoning and CPS. In the present study, all measures can be considered as speeded power tests, which means that rather liberal time restrictions were used to ensure an efficient and pragmatic test administration. However, it may well be that there have been differently strong influences of mental speed in the operationalizations of the three cognitive constructs. In terms of the Brunswik symmetry principle, this also shows that, when choosing operationalizations, not only the level of aggregation and the task contents have to be considered (as it was done in the present study) but also that a symmetrical (fair) comparison is only possible if the operationalizations have comparable time constraints. This issue has received too little attention in previous CPS research and should be considered more strongly in future studies, especially on construct validity.

Third, we partly derived our expectations regarding the associations between working memory, fluid reasoning, and CPS from the assessment of the extent to which the content requirements of the operationalizations are similar (i.e., symmetrical or fair in terms of the Brunswik symmetry principle). Based on the findings of (Süß and Kretzschmar 2018), we assumed that the CPS measure FSYS puts strong demands on figural abilities, to a slightly lesser extent on numerical abilities, and very weak demands on verbal abilities. Furthermore, as we did not consider the difference between the figural and numerical requirements in FSYS to be particularly large and as the relation between numerical and figural abilities is relatively strong, we did not formulate specific expectations regarding the figural and numerical operationalizations. However, our assessment of the content requirements, and thus our expectation, could be disputed because another previous study argued that verbal and not figural or numerical requirements are predominant in FSYS (Wagener 2001). What follows from this is that, in order to choose an appropriate operationalization in terms of the Brunswik symmetry principle and, thus, to derive correct expectations, it is necessary that the requirements of a specific operationalization are known. While this is relatively feasible in the case of working memory and fluid reasoning tasks (see, e.g., Oberauer et al. 2003; Süß and Beauducel 2005), it is more difficult with CPS tasks, since it is in their nature to reflect more complex requirements (Dörner and Funke 2017). For future research, it is therefore important to examine theoretically and empirically which content requirements dominate the respective operationalizations (see, e.g., Kretzschmar et al. 2017).

Fourth, only one, instead of multiple, operationalizations of CPS was used. Thus, generalizations with respect to other CPS operationalizations are only possible to a limited extent (Kretzschmar 2017). The use of different CPS operationalizations in future studies would also reflect the fact that the Brunswik symmetry principle should not only be applied to the predictor side (see left side of Figure 1) but that the symmetry principle should also be considered for the criterion side (see right side of Figure 1). Different CPS operationalizations, ideally with different and distinctive contents (i.e., verbal, numerical, and figural), would allow to investigate different levels of aggregation for the criterion side, as well. An alternative way to consider the Brunswik symmetry principle also on the criterion side would be to consider a more fine-grained scoring of the problem solving processes within one CPS operationalization. For example, there are various approaches in CPS research to evaluate behavioral patterns in order to solve the problem (e.g., exploration or knowledge acquisition strategies); see, (e.g., Greiff et al. 2015; Müller 2013; Wagener and Wittmann 2002), which are located at a lower level of aggregation compared to the commonly used performance scoring (e.g., problem solved or not). Such analyses were outside the scope of the present study but are potentially promising for future studies.

Finally, it has to be emphasized that the present study should be considered as exploratory. The present study, as well as previous studies (e.g., Greiff et al. 2016; Zech et al. 2017), applied secondary data analyses to existing data sets, which were used for related research questions before. This procedure can increase the false positive rate (e.g., Gelman and Loken 2013; Thompson et al. 2020). Therefore, the research question whether working memory plays a unique role in CPS independent of fluid reasoning has to be addressed in further replications studies based on new data sets see (Weston et al. 2019).

## 5. Conclusions

The present study provides little evidence that working memory plays a unique part in solving complex problems independently of fluid reasoning. However, there is a need for further studies on this research question, which particularly take into account the influence of the Brunswik symmetry principle. As exemplified in the present study, the Brunswik symmetry principle is not only crucial with regard to the interpretation of empirical results but can also be useful for study planning. Thus, future studies investigating the interplay of different cognitive abilities will greatly benefit if the Brunswik symmetry principle is considered.

## Figures and Tables

**Table 1:** Descriptive statistics, reliabilities, and correlations.

	(1)	(2)	(3)	(4)	(5)	(6)	(7)	(8)	(9)	(10)	(11)
Working memory											
(1) aggregated	1.00										
(2) verbal	0.33 [0.19,0.47]	1.00									
(3) numerical	0.43 [0.28,0.57]	0.38 [0.22,0.52]	1.00								
(4) figural	0.26 [0.11,0.40]	0.15 [0.00,0.30]	0.28 [0.12,0.44]	1.00							
Fluid reasoning											
(5) aggregated	0.52 [0.41,0.63]	0.25 [0.10,0.39]	0.37 [0.23,0.49]	0.51 [0.39,0.61]	1.00						
(6) verbal	0.25 [0.08,0.40]	0.28 [0.13,0.42]	0.05 [−0.12,0.22]	0.20 [0.04,0.34]	0.33 [0.17,0.47]	1.00					
(7) numerical	0.50 [0.37,0.61]	0.24 [0.10,0.37]	0.43 [0.30,0.55]	0.41 [0.27,0.53]	0.42 [0.27,0.54]	0.21 [0.04,0.36]	1.00				
(8) figural	0.42 [0.29,0.53]	0.04 [−0.11,0.19]	0.32 [0.19,0.44]	0.53 [0.41,0.64]	0.53 [0.41,0.64]	0.35 [0.19,0.50]	0.48 [0.35,0.58]	1.00			
CPS											
(9) total	0.31 [0.13,0.48]	0.16 [−0.03,0.35]	0.20 [0.01,0.38]	0.28 [0.12,0.42]	0.42 [0.25,0.57]	0.15 [−0.01,0.31]	0.38 [0.21,0.54]	0.42 [0.26,0.56]	1.00		
(10) control	0.32 [0.15,0.47]	0.13 [−0.05,0.31]	0.21 [0.02,0.38]	0.32 [0.16,0.46]	0.33 [0.16,0.49]	0.02 [−0.14,0.19]	0.37 [0.20,0.52]	0.36 [0.18,0.50]	0.51 [0.37,0.64]	1.00	
(11) knowledge	0.22 [0.02,0.41]	0.15 [−0.06,0.35]	0.14 [−0.05,0.33]	0.16 [−0.00,0.32]	0.40 [0.20,0.58]	0.24 [0.06,0.42]	0.30 [0.10,0.47]	0.37 [0.20,0.52]	0.51 [0.37,0.64]	0.51 [0.37,0.64]	1.00
*M*	0.00	0.07	0.02	0.04	0.00	0.00	0.00	0.00	0.00	57.59	5.32
*SD*	0.72	0.95	0.98	1.00	0.81	0.71	0.77	0.80	0.87	22.51	1.94
*ω*	0.57	0.88	0.82	0.73	0.74	0.54	0.66	0.72	0.69	0.80	0.41

Note: All aggregated scores and all content-specific fluid reasoning scores were calculated based on z-standardized scores (i.e., with *M* = 0.00 and *SD* = 1.00). Manifest Pearson’s correlations between aggregated scores and sub-scores were corrected for overlap (i.e., part-whole correlation). Ninety-five percent confidence intervals (CI) are stated in brackets.

**Table 2:** Model parameters for different conditions.

	WM Aggregated		WM Verbal		WM Numerical		WM Figural	
**Fluid Reasoning**	**Model 1**	**Model 2**	**Model 1**	**Model 2**	**Model 1**	**Model 2**	**Model 1**	**Model 2**
aggregated	Condition 1		Condition 2		Condition 3		Condition 4	
a	0.65 [0.52;0.77]	0.65 [0.52;0.77]	0.29 [0.12;0.45]	0.29 [0.12;0.45]	0.44 [0.29;0.59]	0.44 [0.29;0.59]	0.65 [0.52;0.78]	0.65 [0.52;0.79]
b	0.53 [0.33;0.71]	0.49 [0.11;0.83]	0.53 [0.32;0.72]	0.52 [0.29;0.72]	0.53 [0.32;0.72]	0.52 [0.27;0.77]	0.52 [0.32;0.71]	0.53 [0.20;0.93]
c		0.05 [−0.33;0.46]		0.02 [−0.20;0.25]		0.01 [−0.26;0.29]		−0.01 [−0.41;0.31]
$R_{adj}^{2}$	0.27 [0.10;0.50]	0.26 [0.11;0.53]	0.27 [0.10;0.51]	0.27 [0.10;0.52]	0.27 [0.10;0.51]	0.27 [0.10;0.53]	0.27 [0.10;0.50]	0.26 [0.10;0.54]
verbal	**Condition 5**		Condition 6		Condition 7		**Condition 8**	
a	**0.33 [0.12;0.53]**	**0.32 [0.11;0.52]**	0.35 [0.17;0.52]	0.35 [0.16;0.52]	0.07 [−0.15;0.28]	0.06 [−0.16;0.27]	**0.28 [0.07;0.47]**	**0.27 [0.07;0.46]**
b	**0.23 [0.01;0.45]**	**0.04 [−0.22;0.28]**	0.21 [−0.01;0.43]	0.14 [−0.09;0.37]	0.21 [−0.01;0.43]	0.18 [−0.05;0.40]	**0.22 [0.00;0.44]**	**0.10 [−0.14;0.33]**
c		**0.41 [0.15;0.66]**		0.14 [−0.12;0.39]		0.24 [0.01;0.48]		**0.33 [0.12;0.55]**
$R_{adj}^{2}$	**0.05 [−0.01;0.20]**	**0.17 [0.03;0.42]**	0.04 [−0.01;0.18]	0.04 [−0.01;0.21]	0.04 [−0.01;0.18]	0.08 [0.00;0.29]	**0.04 [−0.01;0.19]**	**0.13 [0.03;0.33]**
numerical	Condition 9		Condition 10		Condition 11		Condition 12	
a	0.64 [0.49;0.77]	0.63 [0.48;0.77]	0.29 [0.11;0.44]	0.28 [0.11;0.44]	0.53 [0.37;0.67]	0.53 [0.37;0.68]	0.54 [0.38;0.69]	0.53 [0.37;0.69]
b	0.50 [0.30;0.68]	0.39 [0.04;0.77]	0.49 [0.29;0.68]	0.47 [0.25;0.67]	0.49 [0.29;0.67]	0.49 [0.22;0.81]	0.49 [0.29;0.68]	0.42 [0.13;0.70]
c		0.14 [−0.30;0.52]		0.05 [−0.19;0.28]		−0.01 [−0.39;0.31]		0.12 [−0.17;0.40]
$R_{adj}^{2}$	0.24 [0.08;0.46]	0.23 [0.09;0.48]	0.23 [0.08;0.46]	0.23 [0.08;0.46]	0.23 [0.08;0.44]	0.23 [0.08;0.49]	0.24 [0.08;0.45]	0.23 [0.09;0.46]
figural	Condition 13		Condition 14		Condition 15		Condition 16	
a	0.53 [0.38;0.67]	0.52 [0.37;0.66]	0.06 [−0.12;0.24]	0.05 [−0.13;0.23]	0.40 [0.24;0.55]	0.39 [0.24;0.54]	0.68 [0.54;0.81]	0.69 [0.54;0.82]
b	0.54 [0.35;0.71]	0.44 [0.12;0.69]	0.53 [0.34;0.71]	0.52 [0.32;0.70]	0.53 [0.34;0.71]	0.51 [0.27;0.72]	0.52 [0.32;0.70]	0.56 [0.22;0.92]
c		0.16 [−0.15;0.48]		0.18 [−0.03;0.39]		0.04 [−0.21;0.31]		−0.05 [−0.44;0.29]
$R_{adj}^{2}$	0.29 [0.11;0.51]	0.28 [0.13;0.52]	0.28 [0.11;0.50]	0.31 [0.14;0.54]	0.28 [0.11;0.50]	0.27 [0.12;0.51]	0.27 [0.10;0.48]	0.27 [0.11;0.52]

Note: WM = working memory. Model 1 and 2 as displayed in Figure 3. a, b, and c = standardized path coefficients as labeled in Figure 3. $R_{adj}^{2}$ = explained CPS variance adjusted regarding the number of predictors. Ninety-five percent CI are stated in brackets. Conditions highlighted in bold represent conditions in which working memory incrementally explained variance in CPS above and beyond fluid reasoning.

## Data Availability

The data presented in this study are openly available in Open Science Framework at doi:10.17605/OSF.IO/N2JVY.

## Footnotes

1. The link to the Brunswik symmetry principle is not always made explicitly. For example, as the bandwidth-fidelity dilemma (Cronbach and Gleser 1965) is closely related to the Brunswik symmetry principle, studies on this topic can also be interpreted in terms of the Brunswik symmetry principle.

2. As one reviewer correctly pointed out, one could also expect that figural operationalizations should incrementally explain CPS variance above and beyond numerical operationalizations as we assume that the CPS measure put stronger requirements on figural content compared to numerical content. We address this issue in the Discussion section.

3. The present study used the performance scale of FSYS which was most comparable to CPS operationalizations of previous studies. FSYS also provides additional behavior-based scales, some of which are experimental in nature and were of insufficient psychometric quality in the present study (see, Kretzschmar and Süß 2015). Thus, these scales were not considered here but are included in the freely available data set.

4. We have also conducted the analyses with the CPS control performance indicator only (i.e., without the indicator of knowledge acquisition) to examine the robustness of the results. Although the effect sizes (e.g., explained CPS variances) differed, the overall pattern of findings was comparable to that of the aggregated CPS score presented here.

5. The analyses were also performed on the basis of complete data only (i.e., without missing data, *N* = ), which resulted in almost identical results to those presented here.
